# An improved odor bait for monitoring populations of *Aedes aegypti*-vectors of dengue and chikungunya viruses in Kenya

**DOI:** 10.1186/s13071-015-0866-6

**Published:** 2015-04-29

**Authors:** Eunice A Owino, Rosemary Sang, Catherine L Sole, Christian Pirk, Charles Mbogo, Baldwyn Torto

**Affiliations:** International Centre of Insect Physiology and Ecology, P.O BOX 30772-00100 Nairobi, Kenya Africa; Department of Zoology and Entomology, University of Pretoria, Pretoria, South Africa; Centre for Geographic Medicine Research – Coast, KEMRI & KEMRI – Wellcome Trust Research Programme, Kilifi, Kenya

**Keywords:** *Aedes aegypti*, Dengue, Chikungunya, Attractant, Electrophysiology, Mosquito, Traps

## Abstract

**Background:**

Effective surveillance and estimation of the biting fraction of *Aedes aegypti* is critical for accurate determination of the extent of virus transmission during outbreaks and inter-epidemic periods of dengue and chikungunya fever. Here, we describe the development and use of synthetic human odor baits for improved sampling of adult *Ae. aegypti*, in two dengue and chikungunya fevers endemic areas in Kenya; Kilifi and Busia counties.

**Methods:**

We collected volatiles from the feet and trunks of two female and two male volunteers aged between 25 and 45 years. We used coupled gas chromatography- electroantennographic detection (GC/EAD) analysis to screen for antennally-active components from the volatiles and coupled GC-mass spectrometry (GC/MS) to identify the EAD-active components. Using randomized replicated designs, we compared the efficacies of Biogents (BG) sentinel traps baited with carbon dioxide plus either single or blends of the identified compounds against the BG sentinel trap baited with carbon dioxide plus the BG commercial lure in trapping *Ae. aegypti*. The daily mosquito counts in the different traps were subjected to negative binomial regression following the generalized linear models procedures.

**Results:**

A total of ten major EAD-active components identified by GC/MS as mainly aldehydes and carboxylic acids, were consistently isolated from the human feet and trunk volatiles from at least two volunteers. Field assays with synthetic chemicals of the shared EAD-active components identified from the feet and trunk gave varying results. *Ae. aegypti* were more attracted to carbon dioxide baited BG sentinel traps combined with blends of aldehydes than to similar traps combined with blends of carboxylic acids. When we assessed the efficacy of hexanoic acid detected in odors of the BG commercial lure and volunteers plus carbon dioxide, trap captures of *Ae. aegypti* doubled over the trap baited with the commercial BG lure. However, dispensing aldehydes and carboxylic acids together in blends, reduced trap captures of *Ae. aegypti* by ~45%-50%.

**Conclusions:**

Our results provide evidence for roles of carboxylic acids and aldehydes in *Ae. aegypti* host attraction and also show that of the carboxylic acids, hexanoic acid is a more effective lure for the vector than the BG commercial lure.

**Electronic supplementary material:**

The online version of this article (doi:10.1186/s13071-015-0866-6) contains supplementary material, which is available to authorized users.

## Background

Arboviral diseases such as dengue and chikungunya fever transmitted by *Ae. aegypti* are emerging and resurging causing global concern [1,2]. The global incidence of dengue has risen rapidly in recent decades and the disease is now endemic in more than 100 countries in Asia, Africa, and the Americas. Infections from arboviral diseases have also risen and are now estimated at 50–100 million infections every year, with 21,000 fatalities [3]. This puts some 3.6 billion people, that is, half of the world's population, mainly in the urban centers of the tropics and subtropics at risk [4,5]. Cases of chikungunya outbreaks have also increased [6]. In 2004–2005, widespread outbreaks of chikungunya occurred along the Kenyan coast and four island countries in the Indian Ocean including Comoros, Seychelles, Reunion and Mauritius [6,7]. A year later, the outbreak spread to the Indian subcontinent [8] and to south of Italy in 2006 [9]. Outbreaks have also been reported in Central and Latin America as recently as in September 2014 where the epidemic is reported to have overwhelmed hospitals and cut economic productivity [10].

Presently, dengue and chikungunya fevers have no treatment or vaccine [11]. This has left vector control as the only available measure for prevention even though major progress has been made in developing a vaccine against dengue/severe dengue [11]. In addition, disease monitoring for both dengue and chikungunya depends on vector collection and abundance tracking. In our previous work [12], we tested the responses of *Ae. aegypti* to human feet and trunk odors captured in cotton socks and T-shirts in field assays using the Biogents sentinel traps in Busia and Kilifi Counties of Kenya. We found that *Ae. aegypti* responses to the human odors varied with the volunteer and body part and also with the study site. We also analyzed odors from the human volunteers and the BG lure by GC/MS and observed major qualitative differences between the chemical profiles. Aldehydes, fatty acids and ketones dominated human odor profiles, whereas the commercial BG-lure originally comprising of lactic acid, ammonia, and hexanoic acid (caproic acid) [13] released mainly hexanoic acid. Our results suggested that some of the human volunteers who participated in this study could be sources for the identification and development of more potent lures than the BG-lure for *Ae. aegypti.* Here, we report the identification of attractants from human feet and trunk odors for *Ae. aegypti* and field evaluation of improved odor baits for sampling adults of this mosquito species.

## Methods

### Study sites

**Figure 1:**
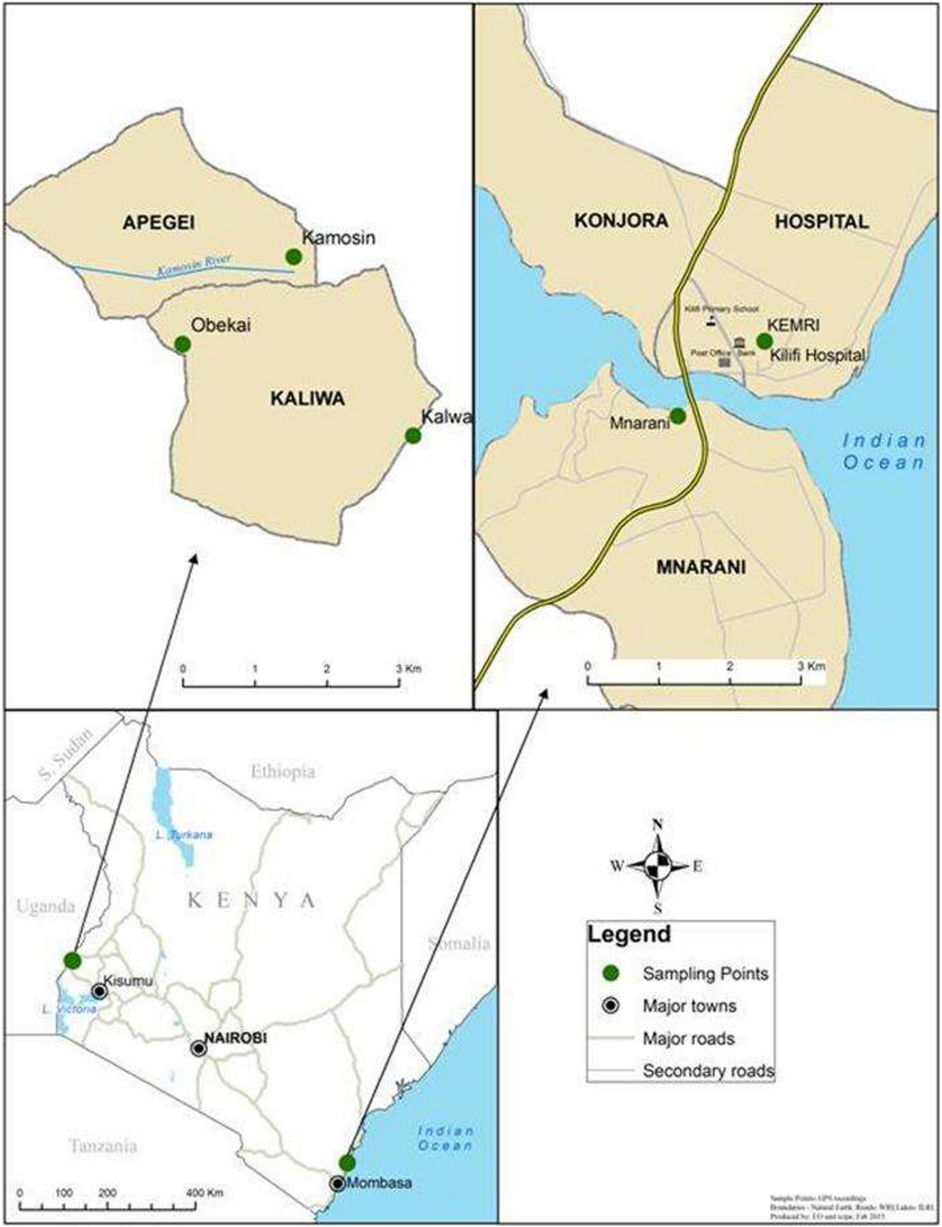
The study sites; Kilifi district in the coast and Busia district in western Kenya.

Field studies were carried out in Kilifi County at the Kenyan coast and Busia County in Western Kenya (Figure 1). An outbreak of dengue was reported in Malindi, Kenya in 1982 [14] and previous seroprevalence studies have shown that dengue infection was prevalent in Malindi area of Kilifi, with chikungunya infection occurring in Busia County [15].

Kilifi County experiences a bimodal kind of rainfall- the long monsoon rains (April-July) and the short rains (October-December) that averages annual rainfall of 950 mm. The temperatures range from a minimum of 21°C and a maximum of 32°C. Busia County on the other hand has an average annual rainfall of 1500 mm. The rainfall pattern is also bimodal; long rains (March-June) and short rains (October-December). Temperatures range from minimum of 14°C and maximum of 30°C.

In Busia County, traps were set up in villages in the rural area namely Obekai (0 30.875 N, 34 12.293 E), Kamosin (0 31.530 N, 34 13.125 E) and Kalwa (0 30.190 N, 34 14.020E). These locations occur at approximately 1189 m above sea level (asl). The main vegetation in these areas consists of large, tall eucalyptus trees that form thick canopies. The local inhabitants are mainly small-scale farmers growing maize, millet and cassava as food crops while a few grow sugarcane and coffee as cash crops. They also keep a few animals mainly cattle, sheep, goats, pigs, chicken and guinea fowls.

In Kilifi county, traps were set up at three sites located in the urban area namely Kenya Medical Research Institute (KEMRI) campus, Kilifi hospital (3 37.800 S, 39 51.483 E) and Mnarani estate (3 38.368 S, 39 50.824 E). These locations occur at approximately 30.5 m asl. The inhabitants in the urban area mainly engage in small businesses or work in offices. They also grow maize, cassava and sweet potatoes and keep a few animals mainly goats.

The traps were set up during the wet seasons at both sites. In Busia, traps were set up in November 2013 and in Kilifi in December 2013 and April 2014.

### Odor collection from trunk and feet of volunteers

Four adult volunteers, 2 males and 2 females, between the ages of 25 and 40 years were identified and enrolled to participate in the study after obtaining informed consent. The two males had participated in our previous study and one of the males was more attractive than the other [12]. The volunteers were each requested to put on clean new cotton T shirts and clean new cotton socks (Lux Industries Ltd 39 K.K Tagarest, Kolkata-700-007) issued to them by the researchers for 18 hrs. The worn socks and T shirts from the volunteers were individually wrapped in at least 4 layers of aluminium foil and stored in cool boxes (10°C) for immediate transportation to the laboratory for odor trapping using the volatile entrainment system as described below.

### Headspace trapping of odors trapped in worn socks and T shirts

The socks and T shirts obtained from the volunteers were held in tightly sealed volatile collection jars (ARS, Gainesville, FL, USA) and odors collected on Super Q adsorbent (30 mg, Alltech, Nicholasville, KY) traps for 24 hr. The Super Q filters were eluted with 150 μl dichloromethane Sigma-Aldrich Corporation (3050 Spruce Street, St. Louis, Missouri 63103 USA) and stored at −80°C until use.

### Mosquitoes

Mosquitoes used in this study were obtained from two different populations; (i) An inbred generation reared at the International Centre of Insect Physiology and Ecology (*icipe*), Duduville campus, Nairobi, established in 2001 from blood-fed and gravid *Ae. aegypti* caught at Rabai, Kilifi County, and (ii) A first filial (F1) generation of *Ae. aegypti* established from eggs collected from Rabai, Kilifi in 2013 and reared in a separate insectary at *icipe’s* Duduville campus. In both cases, *Ae. aegypti* were reared at a mean temperature and relative humidity of day, 28°C, 70% RH and night, 26°C, 80% RH; and a reversed circadian rhythm of light (15:01–3:00) and darkness (3:01–15:00). The newly emerged adult females were maintained on glucose (6% solution *ad libitum*) (Sigma®) continuously available on filter paper and no blood meal. On the experimental days the mosquitoes were deprived of glucose for 6 hrs before the experiments.

### Gas chromatography/electroantennographic detection (GC/EAD)

Volatiles collected from the feet and trunk of volunteers were analyzed by coupled GC/EAD analysis using a Hewlett-Packard (HP) 5890 Series II gas chromatograph equipped with an HP-5 column (30 m × 0.25 mm ID × 0.25 μm film thickness, Agilent, Palo Alto, California, USA). Nitrogen was used as the carrier gas at 1.2 ml/min. Volatiles were analyzed in the splitless mode at an injector temperature of 280°C venting at 0.8 min. The oven temperature was held at 35°C for 5 min, then programmed at 10°C/min to 280°C and maintained at this temperature for 10 min. The column effluent was split 1:1 after addition of make-up nitrogen gas for simultaneous detection by flame ionization detector (FID) and EAD. For EAD detection, silver-coated wires in drawn-out glass capillaries (1.5 mm I.D.) filled with Ringer saline solution [16] served as reference and recording electrodes.

Antennal preparations were made by decapitating 4–7 days old females of *Ae. aegypti* at the base of the head and slicing off the tip of the last antennal segment with a scalpel under a dissecting microscope. The antenna was then mounted on to the micromanipulator such that the base of the head was connected to the reference electrode, and the cut tip of the antenna was connected to the recording electrode. The analog signal was detected through a probe (INR-II, Syntech, Hilversum, the Netherlands), captured and processed with a data acquisition controller (IDAC-2, Syntech, the Netherlands), and later analyzed with soft- ware (EAG 2000, Syntech) on a personal computer. An aliquot (5 μl) of the Super Q-trapped volatile extract from each volunteer’s feet and trunk was analyzed using fresh female antennae in at least three replicate runs.

### Coupled gas chromatography/mass spectrometry (GC/MS)

GC/MS analysis of volatiles was carried out on an Agilent system (Agilent Technologies, Inc., Santa Clara, CA, USA) consisting of a 7890A gas chromatograph, a 5975C Mass spectrometer with a triple Axis detector and an Agilent ChemStation data system. The GC column was an HP-5 MS fused silica capillary (30 m × 0.25 mm × 0.25 μm film thickness) (J&W, Folsom, CA, USA). The carrier gas was helium with a column head pressure of 8.827 psi and flow rate of 1.2 mL/min. Inlet temperature was 270°C and MSD detector temperature was 280°C. The oven temperature was held at 35°C for 5 min and then increased at 10°C/ min to a final temperature of 280°C, which was held for 10.5 min. The identity of each component in the extracts of the volatiles was determined by comparison with references from mass spectral libraries (NIST05, Agilent Technologies [NIST05, Agilent Technologies NIST database, G1033A, revision D.05.01, ChemStation data system (G1701EA, version E.02.00). An aliquot (1 μl) of the volatile extract from each volunteers’ feet or trunk and of synthetic authentic compounds was injected into the GC-MS for analysis.

GC/EAD-active components were identified both by comparing their mass spectral data with those recorded in the Mass Spectral Library NIST 2005 and by co-injection with authentic standards.

### Chemicals

Hexanal, heptanal, hexanoic acid, octanal, nonanal, decanal and undecanal were obtained from (Sigma-Aldrich Chemie (GmbH, Germany) while propionic acid, 3-methylbutyric acid, and 6,10-dimethyl-5,9-undecadien-2-one (geranyl acetone) were sourced from Sigma-Aldrich Corporation (3050 Spruce Street, St. Louis, Missouri 63103 USA). Purities of the compounds ranged between 95% and 99%. The BG lure used in this study was purchased from Biogent, with an expiry date of December 2015. It mainly contains lactic acid, hexanoic acid and ammonia [13].

### Field testing of EAG-active compounds

#### Experiment 1

##### Study design

For field testing in both Kilifi and Busia, mosquitoes were collected using six BG sentinel traps baited with carbon dioxide plus (i) Blend 1; 3-methylbutyric acid and propionic acid each at 0.05 mg/μl at a ratio of 1:1 (ii) Blend 2; nonanal and octanal each at 0.05 mg//μl at a ratio of 1:1(iii) Blend 3; nonanal, octanal, 3-methylbutyric acid and propionic acid each at 0.05 mg/μl dispensed separately at a ratio of 1:1:1:1 (iv) BG-lure (v) worn socks and (vi) worn T shirts.

### Traps baited with human odors

Odors were obtained from the feet and trunk of a male volunteer aged 32 years old in Busia and a male volunteer aged 30 years old in Kilifi. Both of them had donated odors for the GC/EAD tests. The volunteers were requested to put on new, clean, 100% cotton socks and T shirts (Lux Industries Ltd 39 K.K Tagarest, Kolkata-700-007) to trap odors from their feet and trunk for 18 hrs daily for a period of 12 days. New socks and T shirts were provided daily. The volunteers were also provided with odorless soap to bathe with daily and requested to avoid the use of deodorants and perfumes. The socks and T shirts once removed by the volunteers were wrapped in at least 4 layers of aluminium foil and stored in cool boxes at 10°C and transferred into the laboratory and then into −80°C freezer until use. The worn socks and T shirts were used daily to bait BG sentinel traps by hanging them on the rails of the BG sentinel trap inner structure as described in Owino *et al*. [12].

### Traps baited with synthetic chemicals

Preliminary trials to determine the possible range of attractive doses of, nonanal, octanal, propionic acid, 3-methylbutyric acid and hexanoic acid, were conducted in the field at *icipe*’s Nairobi campus. These chemicals were identified as the consistent EAD-active components that were most commonly shared amongst the different volunteers. Concentrations of individual compounds, including 0.005, 0.01, 0.02 and 0.05 mg/μl were evaluated in three replicate trials. Trap captures showed that the optimal attractive dose of nonanal and octanal to *Ae. aegypti* was 0.05 mg/μl while hexanoic acid, 3-methylbutyric acid and propionic acid were effective between 0.01 and 0.05 mg/μl/ [data not shown]. Hexanal and decanal did not show strong attraction to *Ae. aegypti* at the tested concentrations.

To obtain stock concentrations, 100 mg of each EAD-active compound was diluted in 1 ml of hexane. Ten milligrams (10% of the concentration of individual component) of the antioxidant, 2, 6-di-tert-butyl-4-methylphenol (butylated hydroxytoluene, BHT, Aldrich) was then added to the aldehyde stocks to prevent oxidization to their respective corresponding fatty acids. To bait the traps, 50 μl of each compound was transferred from the stock and diluted in hexane to make 100 μl. The solution was adsorbed on cotton wicks measuring 5 mm × 30 mm wrapped in a nylon stocking material measuring 12 mm × 30 mm. The cotton wicks and BG-lure were then inserted into the odor pockets of the BG sentinel traps. Each compound was dispensed from its own cotton wick.

### Mosquito sampling

At each of the study sites, Kilifi and Busia, six different locations were randomly chosen around homesteads. Traps were set up at approximately 100 m away from the nearby house (occupied or unoccupied). The six BG sentinel traps baited as described above were randomly set up at each of the six locations with a distance of at least 100 m between traps. The traps were hung at 0.2 m above the ground and attached to each was a Bioquip igloo that dispensed carbon dioxide in the form of dry ice [12]. To offset any positional bias, traps were rotated every experimental day. The traps were set up at 9.00 am and left to run until 5.00 pm. Trapped mosquitoes were collected and transported to the laboratory where they were freeze-killed and identified under a dissecting microscope to species level using morphological keys [17-19].

### Experiment 2

#### Study design

Comparison of the efficacies of various EAD-active carboxylic acids in attracting *Ae. aegypti* at the two field sites, Kilifi and Busia (Experiment 1), showed that only hexanoic acid strongly attracted this mosquito species. Previously, we had detected it in the odors released from the BG lure (12). It was therefore selected for further evaluation in Kilifi which compared to Busia had a higher density of *Ae. aegypti* (see Results section). This experiment was carried out in five locations in Kilifi. Mosquitoes were collected in the field using five BG sentinel traps baited with carbon dioxide plus either (i) hexanoic acid at 0.05 mg/μl (ii) Blend 2; octanal and nonanal each at 0.05 mg/μl at a ratio of 1:1 (iii) Blend 4; hexanoic acid, nonanal and octanal each at 0.05 mg/μl at a ratio of 1:1:1 (iv) BG-Lure (v) carbon dioxide only. The compounds were dispensed from rubber septa which were inserted into the odor pockets of the BG sentinel traps instead of the cotton wicks wrapped in Nylon materials like in experiment 1. In traps baited with more than one compound, each compound was prepared individually as already described and dispensed separately from rubber septa. The average release rate of the hexanoic acid was 0.7 mg/hr over the 7 hr trapping period [Additional file 1] while the average release rate of hexanoic acid from the BG lure was calculated as 1.9 mg/hr over the same period. [Additional file 2]. The release rates were calculated based on GC/MS peak area comparison with those of authentic standards.

### Mosquito sampling

The five BG sentinel traps were randomly set up at each of the five locations just as described in experiment 1 above after which captured mosquitoes were freeze- killed and identified to species using appropriate keys [17-20].

### Data analysis

The daily mosquito counts in the different traps were subjected to negative binomial regression following the generalized linear models (GLM) procedures in R 3.1.0 [21]. The trap baited with the BG commercial lure was used as the control and the reference category in both field experiments 1 and 2. The incidence rate ratios (IRR), a likelihood measure that mosquito species chose other treatments instead of the reference category, and corresponding P-values were estimated. The Pearson’s chi-square test was applied to evaluate differences between proportions of fed and gravid mosquitoes per treatment trap against the reference category. The tests were performed at 5% significance level.

### Ethics statement

The study was approved by the national ethics review committee based at the Kenya Medical Research Institute (KEMRI) and informed consent was obtained from each of the participants. Different sampling locations were randomly chosen around homesteads after obtaining oral consents from the heads of the homes.

## Results

### GC/EAD and GC/MS analyses of volatiles

**Figure 2:**
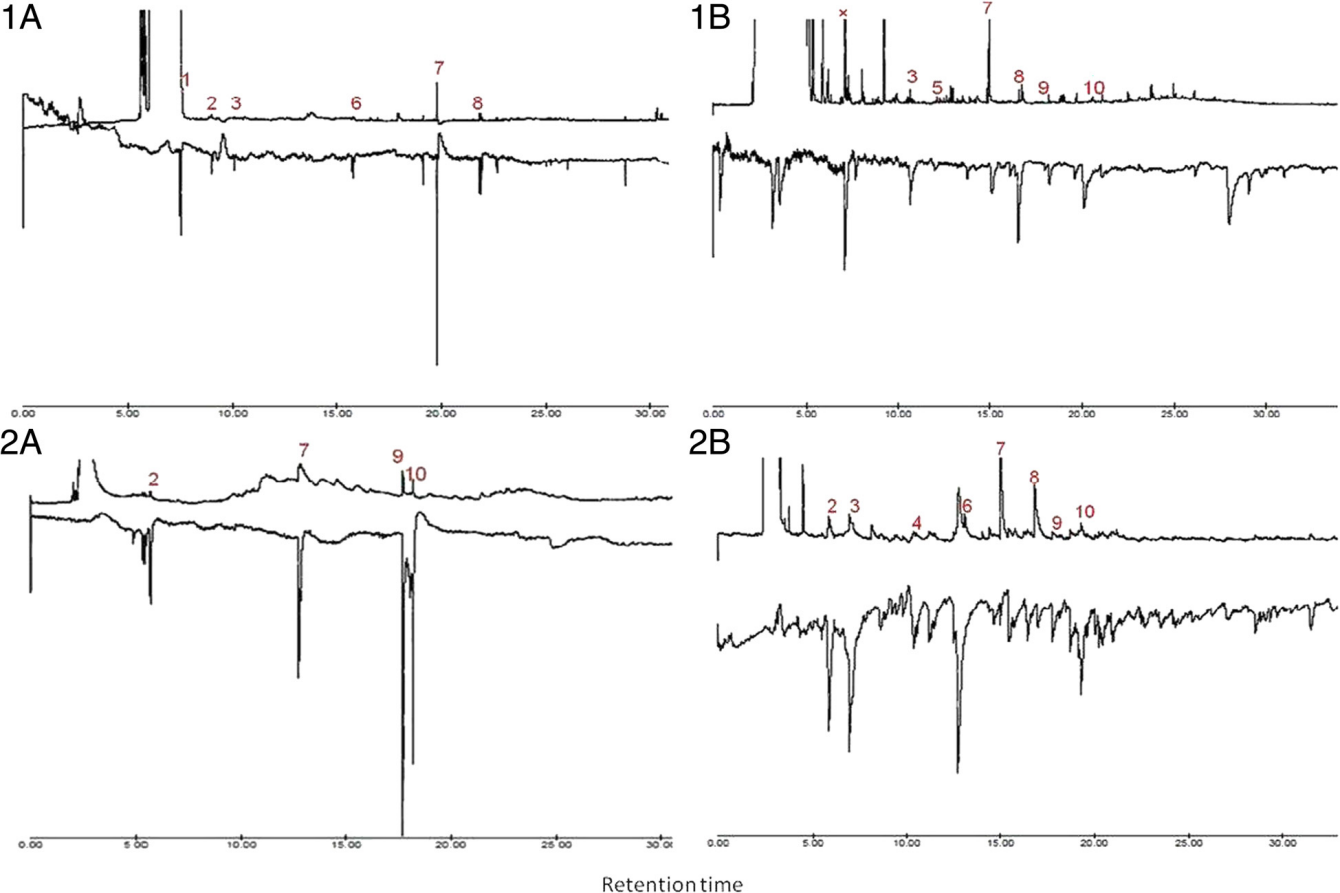
Representative GC/EAD profiles showing EAD- active components identified from; the feet- Panel **1** and trunk - Panel **2**, of volunteers. Panel **A**- GC/EAD responses from F1 generation *Ae. aegypti* from Rabai, Kilifi. Panel **B** – GC/EAD responses from inbred 66^th^ generation *Ae. aegypti* from Rabai, Kilifi. EAD-active components; 1- propionic acid, 2- hexanal, 3- methyl butyric acid, 4- heptanal, 5- hexanoic acid, 6- octanal, 7- nonanal, 8- decanal, 9- undecanal, 10- 6,10-dimethyl-5,9-undecadien-2-one (geranyl acetone).

A total of 21 EAD-active components were identified from the odor collections from the four volunteers, with most of them identified based on selected ion monitoring because they were present in low levels (Table 1). Of these, 10 were common to the trunk and feet odors of at least two of the volunteers consistently eliciting GC/EAD responses from either *Ae. aegypti* obtained from the Rabai, Kilifi F1 generation or the inbred laboratory reared population (Figure 2)*.* Antennal responses were stronger using the F1 generation than the 66^th^ generation of laboratory-reared population of *Ae. aegypti* (Figure 2). The components which consistently elicited EAD activity in odors were identified by GC/MS as the aldehydes; hexanal, heptanal, octanal, nonanal, decanal, undecanal, and the carboxylic acids; propionic acid, 3-methylbutyric acid, hexanoic acid and the ketone, 6,10-dimethyl-5,9-undecadien-2-one (geranyl acetone) (Table 1). Three additional compounds that co-eluted with the solvent (Figure 2, panel 1B) were unidentified. Minor EAD-active components identified from the odors of the different volunteers, were 2-methylbutyric acid, pentanoic acid,1-octen-3-ol, 6-methyl-5-hepten-2-one, 3,7-dimethyl-1-6-octadien-3-ol (linalool), 2-ethylhexanoic acid, [*E*]- or [*Z*]-2-nonenal, nonanoic acid, hexadecanoic acid and octadecanoic acid. Except for 2-nonenal which was identified based on comparison of its mass spectrum with library data, all the other components were identified based on library data and co-injection with authentic standards.

**Table 1:** **Major and** ***m*** **inor GC/EAD active compounds in the volunteers’ feet and trunk odors**

**x**	**GC/M S RT (min)**
Propionic acid (1)	6.2
Hexanal (2)	6.5
3-methylbutyric acid (3)	7.7
Heptanal (4)	9.1
Hexanoic acid (5)	10.8
Octanal (6)	11.2
Nonanal (7)	13.0
Decanal (8)	14.6
Undecanal (9)	16.2
6, 10-dimethyl-5,9-undecadien - 2-one (geranyl acetone) (10)	18.1
**Minor E AD-active compounds**	
2-methybutyric acid	7.9
Pentanoic acid	8.7
1-octen - 3- ol	10.8
6-methyl-5-hepten-2-one	10.9
3,7-dimethyl- 1,6-octadien- 3-ol (linalool)	13.0
2-ethylhexanoic acid	13.3
[*E*] or [Z]-2-noneral	14.0
Nonanoic acid	15.5
Dodecanal	17.5
Hexadecanoic acid	23.5
Octadecanoic acid	25.6

RT-retention time.

#### Field tests

##### Experiment 1

**Figure 3:**
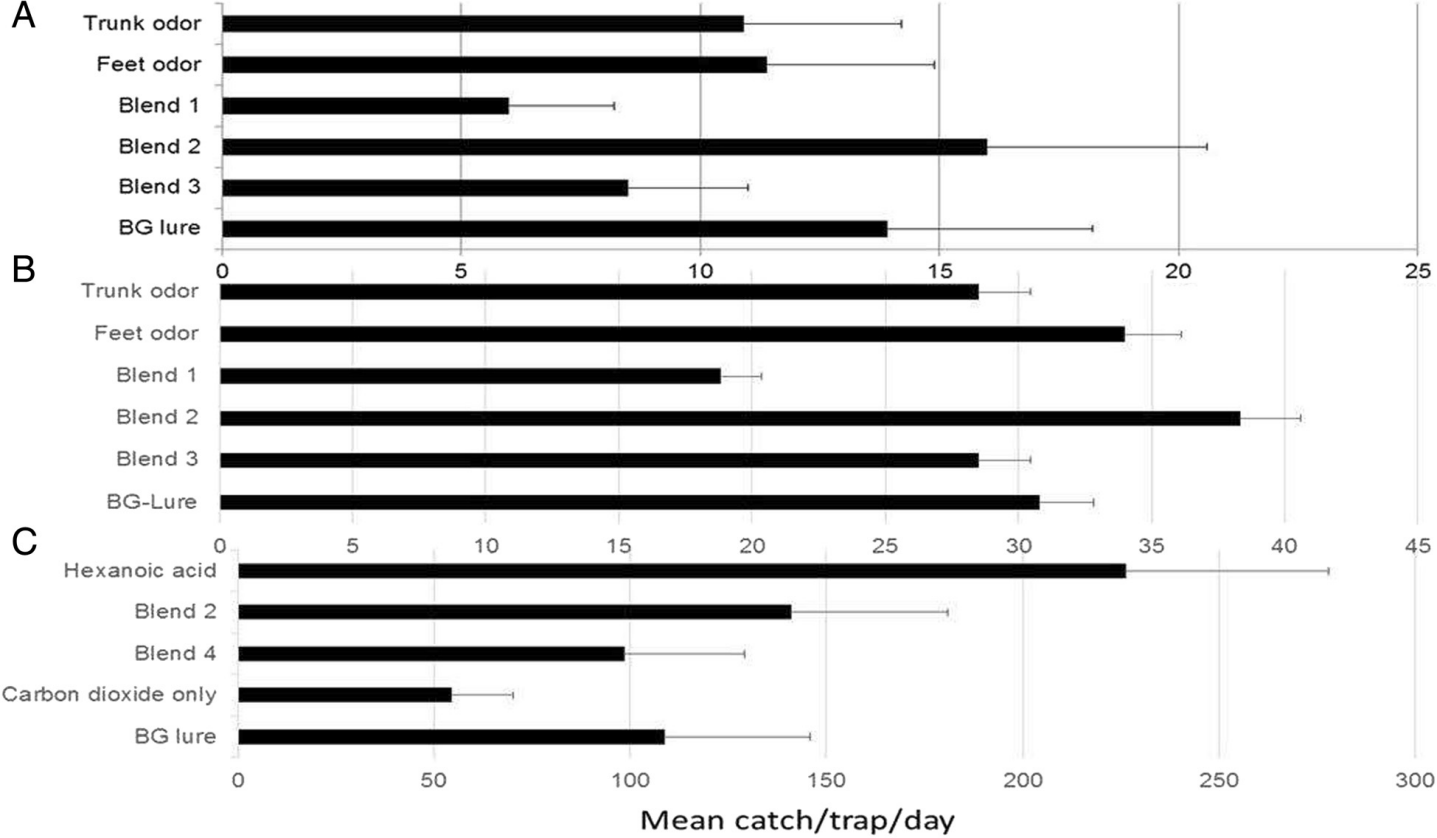
The mean number ± S.E of *Aedes aegypti* captured by the various BG sentinel traps baited with different baits in Busia and Kilifi County. Blend 1; Acids - propionic + 3-methylbutyric acid, Blend 2; Aldehydes - nonanal + octanal, Blend 3; Blend 1 + Blend 2, Blend 4; Blend 2 + hexanoic acid. The different panels show comparisons at the two locations; Panel **A** - Experiment 1 in Busia, Panel **B** - Experiment 1in Kilifi and Panel **C** - Experiment 2 in Kilifi. Error bars indicate standard error of the mean.

Of the total 2,954 *Ae. aegypti* captured, a significant number (~3-fold more) was captured in Kilifi (n = 2,153) than in Busia (n = 801) [p < 0.001]. The trap baited with the binary aldehyde blend of nonanal and octanal (Blend 2) plus carbon dioxide captured 1.3-fold more *Ae. aegypti* than similar traps baited with the BG commercial lure [IRR = 1.3, 95% CI: 0.61-2.75, p = 0.49] (Figure 3). In contrast, the trap baited with the binary carboxylic acid blend comprising 3-methylbutyric acid and propionic acid (blend 1) plus carbon dioxide- captured only 0.6-fold of *Ae. aegypti* compared to captures by the trap baited with the commercial BG lure. However, when the aldehydes and the carboxylic acids were dispensed together (Blend 3), there was a 45% reduction in trap captures [IRR = 0.75 95% CI: 0.43- 1.55 p = 0.40] (Table 2). The same trap capture pattern was found in Kilifi where the overall order of trap performance was Blend 2 (nonanal+octanal) > volunteer 2 feet odors > BG lure > volunteer 2 trunk odors = Blend 3 (nonanal + octanal +3-methylbutyric acid + propionic acid) > Blend 1(3-methylbutyric acid + propionic acid) (Table 2).

**Table 2:** **Comparisons of** ***Ae. aegypti*** **captured by BG sentinel traps baited with different odor baits relative to the control (BG sentinel trap baited with the BG commercial lure) in Experiment 1 in Busia and Kilifi**

**Site**	**Treatment**	**IRR (95%CI)**	**P value**	**Site**	**Treatment**	**IRR (95%CI)**	**P value**
Busia	Blend 3	0.75(0.43-1.55)	0.40	Kilifi	Blend 3	0.91(0.35-2.41)	0.858
Busia	Blend 2	1.3(0.61-2.75)	0.49	Kilifi	Blend 2	1.23(0.47-3.24)	0.665
Busia	Blend 1	0.62(0.29-1.35)	0.23	Kilifi	Blend 1	0.61(0.23—1.6)	0.307
Busia	Volunteer 1 feet odors	1.12(0.53-2.4)	0.76	Kilifi	Volunteer 2 feet odors	1.09(0.42-4-2.8)	0.849
Busia	Volunteer 1 trunk odors	1.01(0.48-2.15)	0.97	Kilifi	Volunteer 2 trunk odors	0.91(0.35—2.41)	0.858

Estimated incidence rate ratio (IRR); confidence interval (CI) and corresponding P-values based on comparison to the control (BG lure baited trap) following generalized linear model (GLM) with negative binomial error structure and log link in R 3.1.0 software. The IRR for the control is 1; values above this indicate better performance while values below indicate under performance relative to the control. Blend 1; propionic acid and 3-methylbutyric acid, Blend 2; nonanal + octanal. Blend 3; Blend 1 + Blend 2.

##### Experiment 2

In the second study carried out in Kilifi which was carried out based on the results from Expt. 1, whereby *Ae. aegypti* was found to be more abundant than in Busia, a total of 6, 239 *Ae. aegypti* were trapped. The trap baited with carbon dioxide and hexanoic acid captured 2.2-fold more *Ae. aegypti* than the trap baited with carbon dioxide and the BG lure [IRR = 2.2, 95% CI: 0.82-5.87, p = 0.109] (Figure 2). However, similar traps baited with hexanoic acid dispensed together with nonanal and octanal, only captured 0.95-fold more *Ae. aegypti* than traps baited with the BG lure (Table 3) showing a 50% reduction of trap captures relative to captures by the hexanoic acid baited trap. The hexanoic acid baited trap also captured more *Ae. aegypti* than all the other traps, with trap performance in the order of hexanoic acid > Blend 2 (nonanal + octanal) > BG–lure > blend 4 (hexanoic acid and nonanal + octanal) > carbon dioxide only (Table 3). Comparison of trap captures showed a significantly higher proportion of female than male *Ae. aegypti* in all the traps (Table 4). A further comparison of captures for fed and gravid mosquitoes per trap showed that the trap baited with hexanoic acid and carbon dioxide captured significantly higher proportions of fed p = 0.047 *Ae. aegypti* than the BG commercial lure plus carbon dioxide baited trap. It also captured 1.2-fold more gravid *Ae. aegypti* than the trap baited with the BG lure (Table 4).

**Table 3:** **Comparisons of** ***Ae. aegypti*** **trapped by BG sentinel traps baited with different odor baits relative to the control (BG sentinel trap baited with the BG commercial lure) in Experiment 2 in Kilifi County**

**Site**	**Treatment**	**IRR (95%CI)**	**P value**
Kilifi	Carbon dioxide only	0.57(0.21 – 1.52)	0.255
Kilifi	Blend 2	1.33 (0.50 -3.57)	0.552
Kilifi	Blend 4	0.95(0.36- 2.56)	0.931
Kilifi	Hexanoic acid	2.2 (0.82- 5.87)	0.109

Estimated incidence rate ratio (IRR); confidence interval (CI) and corresponding P-values based on comparison to the BG lure following generalized linear model (GLM) with negative binomial error structure and log link in R 3.1.0 software. The IRR for the control is 1; values above this indicate better performance while values below indicate under performance relative to the control.

**Table 4:** **Comparisons of** ***Ae. aegypti*** **catch percentages per trap by sex and abdominal status with corresponding p values and catch indices (CI) in Experiment 2**

**Bait/Mosquito count**	**BG Lure**	**No bait**	**Blend 1**	**Blend 2**	**Hexanoic acid**
**Total**	1028	587	985	1377	2262
♀ **Percentage**	55.4	61.8	60	67.4	54.2
♂ **Percentage**	44.6	38.2	40	32.6	45.8
**Fed percentage**	0.9	0.3	0.9	1.5	4.9
**CI**	1	0.3	1	1.6	5.4
**P-value**	-	1	1	1	0.047*
**Gravid percentage**	4.7	0	0.7	3.2	5.8
**CI**	1	0	0.15	0.7	1.23
**P-value**	-	0.11	0.07	0.804	0.97

Catch percentages, Catch indices (CI) and corresponding p values. Asterisks on p values indicate significant difference of the catch percentage with the catch percentage of the control (trap baited with the BG commercial lure). The P-values are based on pair-wise comparison following chi-square goodness-of-fit in R 3.1.0 software.♂-Male *Ae. aegypti*, ♀- female *Ae. aegypti*.

## Discussion

This study investigated volatiles released from the feet and trunk of human volunteers and isolated predominantly aldehydes and carboxylic acids as the electrophysiologically-active components using antennae of *Ae. aegypti*. Electrophysiological activity for some of these aldehydes and carboxylic acids from human skin odors have previously been reported for various mosquito species. For example octanal and nonanal, identified from the human feet, trunk and armpit were reported to elicit electrophysiological response in antennae of *Ae. aegypti* [22,23] and *Aedes mcintoshi* [24], a major vector of Rift Valley fever virus, and *Culex quinquefasciatus* [25], the major vector of West Nile virus in bird headspace volatiles, respectively. Carboxylic acids were reported to elicit EAG responses in *Ae. aegypti* [22,23], *An. gambiae* [26] and *Cx. quinquefasciatus* [27]. These findings emphasize the importance of aldehydes and carboxylic acids in host seeking behavior of *Ae. aegypti.* Both aldehydes and carboxylic acids have previously been reported as common residues on human skin [28,29]. They play a vital dose dependent role in the balance of attraction and inhibition to host seeking *Ae. aegypti* [28-30]. For example, individuals with relatively higher concentrations of aldehydes, especially nonanal, were less attractive to *Ae. aegypti*. [30,31]. Apart from their role in host seeking behavior, some of the identified carboxylic acids like hexadecanoic acid and octadecanoic acid have shown significant concentration dependent positive response to oviposition by *Ae. aegypti* after extraction from conspecific eggs [32,33].

Also, identified as EAG-active in the present study were two ketones; 6, 10-dimethyl-5, 9-undecadien-2-one (geranyl acetone) and 6-methyl-5-hepten-2-one, and two alcohols; 1-octen-3-ol and 3, 7-dimethyl-1-6-octadien-3-ol (linalool), reported previously to elicit electrophysiological activity in *Ae. aegypti* and to have a concentration dependent attractant and repellency effects on this mosquito species [34,35]. Notably, these EAG-active components varied between volunteers, and also varied between body parts with carboxylic acids detected mainly in the feet odors while aldehydes were dominant in the trunk odors. Qualitative differences in odors released between different individuals and also from their body parts have been reported previously [12,22]. The origin of human-specific volatiles emanating from different body regions has been attributed to the aggregation of diverse communities of micro biota [34-38], which differ both in quality and quantity between different individuals and are responsible for driving the attraction of mosquitoes to different host individuals [36,39]. Although it was apparent that there was no difference in the EAD-active compounds that both the F1 and the inbred generations of *Ae. aegypti* detected, antennae of the F1generation detected the compounds more strongly than the inbred population. This suggests that inbreeding may lead to partial loss of antennal sensitivity in agreement with the findings using tsetse fly antennae to isolate EAD-active compounds from odors of vertebrate hosts [40].

In the field evaluation of odors, we found that traps baited with the binary blend comprising octanal and nonanal, each dispensed at 0.05 mg/μl, captured more *Ae. aegypti* than all the other traps including the traps baited with natural human odors (worn socks and worn T-shirts) in both Busia and Kilifi. Similar results showing high attractiveness of aldehydes to mosquitoes have been reported before where a bait formulated from four aldehydes (heptanal, octanal, nonanal and decanal) combined with CO_2_ doubled to tripled trap captures of a CDC trap without a light bulb compared to a control trap baited with CO_2_ alone [24]. It has also been reported that traps baited with nonanal alone significantly captured more *Cx. quinquefasciatus* than traps baited with no odors [25]. Together, these results greatly improve upon our knowledge of odor-based technologies for trapping mosquitoes [23,41] and represent a significant advancement in attempts to develop synthetic lures, which would effectively compete against humans for host seeking mosquitoes in field settings. They also suggest that it is possible to formulate synthetic odor blends that are highly attractive to *Ae. aegypti* without including all the physiologically-active components found in natural human odors. Thus, odor baits may represent a future potential control tool for mass trapping to reduce vector population around houses in disease endemic villages.

An interesting observation that we made in the field based on our trap captures was that of antagonism that appeared to result into a spatial repellency effect between aldehydes and carboxylic acids when dispensed side by side. This was in sharp contrast to our expectations that trap captures with a blend of, the attractive carboxylic acid, hexanoic acid and the attractive binary aldehyde blend of nonanal and octanal was 45% less attractive than that of hexanoic acid alone. Similar antagonistic and spatial repellent effect on mosquitoes by synthetic human odor blends have been observed before however, mainly in laboratory assays [34,42]. For example, it was observed that linalool when used alone, attracts mosquitoes to a trap; however, when used with CO_2_, or with l-octen-3-ol, both of which are mosquito attractants on their own [43], reduced mosquito collection size by as much as 50% [42]. It could also be argued that the carboxylic acids especially hexanoic acid and the aldehydes (nonanal and octanal) are attractants on their own but act as inhibitors when combined with each other. These findings are in line with a previous study that observed that in the absence of gaseous lactic acid, N, N-diethyl-meta-toluamide (DEET) attracted mosquitoes but when mixed with the already attractive lactic acid, DEET reduced mosquito captures [44]. Evidently, the presence of the inhibitor severely impedes the ability of mosquitoes to detect odors that would normally be highly attractive.

We also observed that in Experiment 2, carried out in Kilifi, traps baited with hexanoic acid and carbon dioxide captured more mosquitoes than any other trap including the trap baited with carbon dioxide and the BG commercial lure which also contains hexanoic acid as one of its components [13]. This difference could be associated with the different concentrations and release rates of hexanoic acid in our bait compared to that of the BG lure. The release rate of our trap baited with hexanoic acid was 0.7 mg /hr, ~3- fold less than that released by the BG lure at 1.9 mg/hr. Previous studies report that the effectiveness of hexanoic acid depends on its release rate. For example, at 0.3 ml/min, hexanoic acid had little effect on the attractiveness of lactic acid while increasing it to 100-fold at 30 ml/min, significantly increased attraction of lactic acid to *Ae. aegypti*. At a 1000-fold increase, 300 ml/min, caused a significant decline in attraction [45]. These results are in line with our previous findings where we observed that human trunk and feet odors that were more attractive to *Ae. aegypti* than the BG commercial lure in field bioassays contained between 2-18-fold less hexanoic acid than that present in the BG commercial lure [12].

Our results also showed that in Experiment 2, traps baited with carbon dioxide and hexanoic acid captured higher proportions of blood fed, gravid and male *Ae. aegypti* than all the other traps. This makes hexanoic acid a superior bait in the surveillance and monitoring of these arbovirus vectors. Blood fed mosquitoes provide information on the interactions between host, vector and possible reservoirs, and helps to identify and evaluate the role of potential bridge vector species in the transmission of pathogens of public health importance [46]. They also give information regarding the feeding preference, seroconversion status of that host, and infectivity level of the reservoir host, [47] which immensely helps researchers to understand the ecology of arboviruses spread by mosquitoes. Gravid mosquitoes on the other hand are a high priority in arboviral surveillance programs because they are likely to be already exposed to virus infection through previous feeding, hence are likely indicators of virus activity [48]. Furthermore, hexanoic acid has been reported to be an effective attractant of gravid *Ae. aegypti* that could potentially be used as an oviposition pheromone to lure gravid mosquitoes to traps laced with the killer insecticide, Temephos in a ‘lure to kill’ vector control technique [49]. Lastly, it has been established that although male *Ae. aegypti* are not blood feeders they can be infected with dengue and chikungunya viruses via transovarial transmission [50,51]. They can also transmit the viruses venereally to the females who can then transmit it to humans [52]. Therefore, a trap that is more efficient in capturing male *Ae. aegypti* would be more helpful in dengue and chikungunya fever control programs.

## Conclusions

We conclude that natural human skin odor is a good source for identifying attractant compounds that could be used to improve the existing commercial lures for effective surveillance of *Ae. aegypti* in the field. However, blend composition and release rate are critical to determining vector behavioral response. It is clear that some compounds such as hexanoic acid when released alone at a slow rate are an effective lure to *Ae. aegypti* than when released in blends or at higher rates. Future work should therefore focus on the areas of release rates and effective formulation of hexanoic acid combined with carbon dioxide as a potent lure for monitoring populations of *Ae. aegypti.*

## Additional files

Additional file 1:
**Average release rate of hexanoic acid in the developed odour attractant.**
Additional file 2:
**Average release rate of hexanoic acid in the BG-Lure.**
