# Supplementary material for: An improved odor bait for monitoring populations of Aedes aegypti-vectors of dengue and chikungunya viruses in Kenya
Source: Parasit Vectors. 2015 Apr 29;8:253. doi: 10.1186/s13071-015-0866-6 (PMC4418051; doi:10.1186/s13071-015-0866-6)
Supplement: Additional file 1: — Average release rate of hexanoic acid in the developed odour attractant. [file 13071_2015_866_MOESM1_ESM.docx]

**Additional file 1. Average release rate of hexanoic acid in the developed odour attractant**

| Hexanoic acid | Time interval | Peak Area | Release rate in mg/hr |
| --- | --- | --- | --- |
|  | 00:30 | 1,764,697,680 | 0.000463674 |
|  | 01:30 | 1,831,809,120 | 0.000480452 |
|  | 02:30 | 3,039,432,410 | 0.000782358 |
|  | 03:30 | 3,776,438,300 | 0.00096661 |
|  | 04:30 | 2,276,606,700 | 0.000591652 |
|  | 05:30 | 3,223,380,340 | 0.000828345 |
|  | 06:30 | 2,845,563,960 | 0.000733891 |
| Average |  | 2,679,704,073 | 0.000692426 |
