# Supplementary material for: An improved odor bait for monitoring populations of Aedes aegypti-vectors of dengue and chikungunya viruses in Kenya
Source: Parasit Vectors. 2015 Apr 29;8:253. doi: 10.1186/s13071-015-0866-6 (PMC4418051; doi:10.1186/s13071-015-0866-6)
Supplement: Additional file 2: — Average release rate of hexanoic acid in the BG-Lure. [file 13071_2015_866_MOESM2_ESM.docx]

**Additional file 2. Average release rate of hexanoic acid in the BG-Lure**

| BG lure | Time interval | Peak Area | Release rate in g/hr | Release rate mg/hr |
| --- | --- | --- | --- | --- |
|  | 00:30 | 8,165,169,801 | 0.002063792 | 2.06379245 |
|  | 01:30 | 7,719,027,698 | 0.001952257 | 1.952256925 |
|  | 02:30 | 9,439,653,549 | 0.002382413 | 2.382413387 |
|  | 03:30 | 5,968,987,884 | 0.001514747 | 1.514746971 |
|  | 04:30 | 8,398,191,658 | 0.002122048 | 2.122047915 |
|  | 05:30 | 5,554,863,859 | 0.001411216 | 1.411215965 |
|  | 06:30 | 7,540,982,408 | 0.001907746 | 1.907745602 |
|  | Average |  | 0.001907746 | 1.907745602 |
|  |  |  |  | 1.907745602 |
